# Deep Learning Prediction Boosts Phosphoproteomics-Based Discoveries Through Improved Phosphopeptide Identification

**DOI:** 10.1016/j.mcpro.2023.100707

**Published:** 2023-12-26

**Authors:** Xinpei Yi, Bo Wen, Shuyi Ji, Alexander B. Saltzman, Eric J. Jaehnig, Jonathan T. Lei, Qiang Gao, Bing Zhang

**Affiliations:** 1 Lester and Sue Smith Breast Center, Baylor College of Medicine, Houston, Texas, USA; 2 Department of Molecular and Human Genetics, Baylor College of Medicine, Houston, Texas, USA; 3 Department of Liver Surgery and Transplantation, Liver Cancer Institute, Zhongshan Hospital and Key Laboratory of Carcinogenesis and Cancer Invasion of the Ministry of China, Fudan University, Shanghai, China; 4 Mass Spectrometry Proteomics Core, Advanced Technology Cores, Baylor College of Medicine, Houston, Texas, USA

**Keywords:** phosphoproteomics, deep learning, rescore, phosphosite localization, liver cancer, retention time, fragment ion intensity, EGFR, phosphopeptide identification

## Abstract

Shotgun phosphoproteomics enables high-throughput analysis of phosphopeptides in biological samples. One of the primary challenges associated with this technology is the relatively low rate of phosphopeptide identification during data analysis. This limitation hampers the full realization of the potential offered by shotgun phosphoproteomics. Here we present DeepRescore2, a computational workflow that leverages deep learning-based retention time and fragment ion intensity predictions to improve phosphopeptide identification and phosphosite localization. Using a state-of-the-art computational workflow as a benchmark, DeepRescore2 increases the number of correctly identified peptide-spectrum matches by 17% in a synthetic dataset and identifies 19% to 46% more phosphopeptides in biological datasets. In a liver cancer dataset, 30% of the significantly altered phosphosites between tumor and normal tissues and 60% of the prognosis-associated phosphosites identified from DeepRescore2-processed data could not be identified based on the state-of-the-art workflow. Notably, DeepRescore2-processed data uniquely identifies EGFR hyperactivation as a new target in poor-prognosis liver cancer, which is validated experimentally. Integration of deep learning prediction in DeepRescore2 improves phosphopeptide identification and facilitates biological discoveries.

## Highlights

• DeepRescore2 leverages deep learning to improve phosphopeptide identification.
• Demonstrated sensitivity and accuracy in synthetic and biological datasets.
• Increased identification of prognostic phosphosites in liver cancer.
• EGFR hyperactivation as a new target in poor-prognosis liver cancer.

## Graphical Abstract

**Figure:**
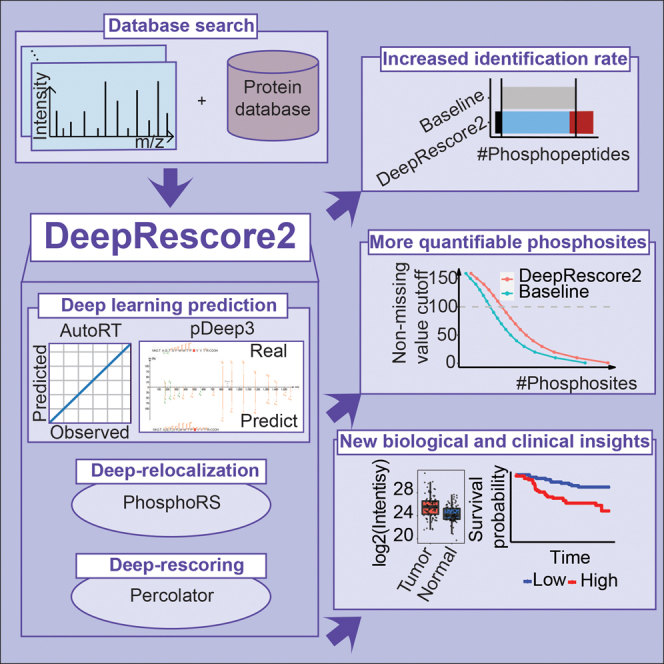

## In Brief

Shotgun phosphoproteomics enables high-throughput analysis of phosphopeptides in biological samples, but low phosphopeptide identification rates in data analysis limit its potential. We introduce DeepRescore2, a computational workflow using deep learning-based retention time and fragment ion intensity predictions to improve phosphopeptide identification and phosphosite localization. We benchmark DeepRescore2 against existing workflows on a synthetic phosphopeptide dataset and apply it to real-world biological datasets, revealing improved sensitivity, fewer missing values, and enhanced phosphoproteomics-based biological discoveries.

Post-translational modifications (PTMs) are ubiquitous in human cells and are broadly involved in the regulation of protein activity, localization, stability, and interaction. One of the most important and extensively studied PTMs is phosphorylation, which plays a critical role in regulating a wide range of biological processes and signaling pathways (1). Shotgun phosphoproteomics, in which proteins are digested by proteases such as trypsin and analyzed *via* liquid chromatography-tandem mass spectrometry (LS-MS/MS), provides an unbiased, high-throughput method to systematically study phosphorylation in biological samples (2).

The first and absolutely essential step in phosphoproteomics data analysis is the identification of phosphopeptides based on MS/MS spectra, which is commonly achieved through database searching (3). Multiple tools, such as MaxQuant (4), MS-GF+ (5), X!Tandem (6), Comet (7), Mascot (8), pFind (9), SEQUEST (10), and MSFragger (11), are available for this analysis. The peptide-spectrum match (PSM) scoring algorithms employed by these search engines are primarily focused on matching spectra to potential peptides or phosphopeptides without providing a formal assessment of the confidence regarding the localization of phosphorylation sites. To ascertain the confidence level associated with each potential phosphorylation site candidate in an identified peptide sequence, various computational algorithms have been developed. Mascot Delta score (12) and SLIP (13) compute the difference of PSM scores associated with different competing phosphosites to determine site localization. PhosSA (14) uses a dynamic programming algorithm for phosphorylation site assignment. Many other algorithms employ probability-based scores to determine phosphorylation site localization, such as AScore (15) and phosphoRS (16) implemented in PeptideShaker (17), PTMScore (18) implemented in MaxQuant, and PTMprophet (19) implemented in philosopher (20).

Despite the availability of numerous computational tools, phosphopeptide identification remains a challenging task, as clearly indicated by the high level of discrepancy among identification results generated by different computational tools on the same data (21). Because each peptide sequence may include multiple candidate phosphorylation sites, the search space in phosphoproteomics is much larger compared with an unmodified search, and this can lead to reduced sensitivity and increased false positive rate in phosphopeptide identification and phosphosite localization. Phosphosite localization is further hampered by the lack of site-determining ions in many experimental spectra. As a result, the spectrum identification rate in a phosphoproteomics experiment is much lower than that in a global proteomics experiment (Supplemental Fig. S1), limiting the potential of phosphoproteomics-based biological discoveries. Moreover, a low spectrum identification rate in individual experiments further leads to many missing values in the resulting dataset, which further reduces the statistical power for biological discoveries.

Recent advancements in deep learning have provided new opportunities for proteomics (22). Deep learning–derived features, such as the retention time (RT) difference between experimentally observed and computationally predicted RTs and the similarity between experimentally observed and computationally predicted MS/MS spectra have been shown to effectively discriminate correct and incorrect PSMs in phosphoproteomics, and these features have been used as evaluation metrics to benchmark computational tools for phosphopeptide identification and phosphosite localization (21). Incorporating these features into PSM rescoring has been shown to improve peptide identification in global proteomic profiling and immunopeptidomic profiling (23, 24, 25, 26, 27). Recently, it has been shown that deep learning-based fragment ion intensity prediction can facilitate phosphorylation site localization (28). However, the potential utility of retention time prediction in phosphorylation site localization has not been investigated. Furthermore, there is no method combining deep learning-facilitated PSM rescoring and site localization to improve the sensitivity and accuracy of phosphopeptide identification.

Built upon our previously published DeepRescore tool that uses deep learning to improve peptide identification in immunopeptidomics (23), we present in this paper DeepRescore2, a computational workflow that leverages deep learning-based fragment ion intensity prediction and RT prediction for phosphopeptides to enhance phosphosite localization and PSM rescoring. We benchmark DeepRescore2 against existing state-of-the-art workflows for phosphosite localization and PSM rescoring on a synthetic phosphopeptide dataset. We also demonstrate its application to three real-world biological datasets to improve the sensitivity of phosphopeptide identification, reduce the number of missing values, and boost phosphoproteomics-based biological discoveries.

## Experimental Procedures

### Datasets

One LC-MS/MS dataset of synthetic phosphopeptides and three real-world LC-MS/MS phosphoproteome datasets from biological samples were used in this study. Raw MS data of the synthetic dataset were downloaded from the PRIDE database (https://www.ebi.ac.uk/pride/) with the accession key PXD000138, which included 96 libraries generated from 96 seed peptides (29). For each seed peptide, the seed position was synthesized with either serine, threonine, or tyrosine or their phosphorylated forms, and the amino acids before or after the seed position were permuted with 20 amino acids to generate up to 2400 different phosphorylated or non-phosphorylated peptides for each library. In this study, we used libraries 1 to 11, which were generated within a single day (Supplemental Table S1). A PSM was considered correct if the identified peptide sequence and phosphorylation site localization matched any of the phosphorylated peptides listed in the matched library (21, 29). One of the three biological datasets was a label-free phosphoproteome dataset, and the other two were tandem mass tag (TMT) phosphoproteome datasets. The label-free dataset was from the U2OS cell line. Raw data were downloaded from the PRIDE database with the accession key PXD023665 (30), and three raw files were used in this study for method evaluation (Supplemental Table S1). Raw data of the TMT datasets were downloaded from the Proteomic Data Commons (PDC, https://pdc.cancer.gov/pdc/). The first one is a TMT10-labeled phosphoproteome dataset from the National Cancer Institute’s Clinical Proteomic Tumor Analysis Consortium (CPTAC) uterine corpus endometrial carcinoma (UCEC) study (31) with the accession key PDC000126, and the first plex including 12 fractions were used in this study for method evaluation (Supplemental Table S1). The second dataset is a TMT10-labeled phosphoproteome dataset from the International Cancer Proteogenome Consortium (ICPC) Hepatitis B Virus (HBV)-related hepatocellular carcinoma (HCC) study (32) with the accession key PDC000199, and all 33 plexes were used in this study for biological discovery (Supplemental Table S1). All the raw MS/MS data files used were converted to MGF files using ProteoWizard (v3.0.19014).

### Database Searching

For the synthetic dataset, we followed the original study (29) and used MaxQuant (v1.6.5.0) to search the MS/MS data against the human IPI v3.72 database concatenated with the default MaxQuant contaminant database. Oxidation of methionine (+15.9949) and phosphorylation of serine, threonine and tyrosine (+79.9663) were used as variable modifications, and no fixed modification was used. Trypsin/P was used as the digestion enzyme and up to 4 missed cleavages were allowed. The default settings for other parameters were used, but with all statistical filters in MaxQuant, including PSM, protein, and site level false discovery rates (FDRs), disabled in order to collect all possible candidate PSMs for downstream analyses.

For the U2OS and UCEC datasets, MS-GF+ (v2019.02.28) (5), Comet (2018.01 rev.4) (7), X!Tandem (v2017.2.1.2) (6), and MaxQuant (v1.6.5.0) (4) were used to evaluate the performance of DeepRescore2 in combination with different search engines. For the HCC dataset, MaxQuant (v1.6.5.0) was used as the search engine. These datasets were searched against the human protein database downloaded from Uniprot (v20190214) and concatenated with the default contaminant database in the MaxQuant analysis. Parameters for database searching were set as follows: Oxidation of methionine (+15.9949) and phosphorylation of serine, threonine, and tyrosine (+79.9663) were used as variable modifications, and carbamidomethylation of cysteine (+57.02146) was used as fixed modification. For the TMT datasets, TMT labeling (+229.1629) of peptide N-termini and lysine residues were specified as fixed modifications. Trypsin/P was used as the digestion enzyme. Up to 2 missed cleavages were allowed for the U2OS and HCC data, and up to 4 missed cleavages were allowed for the UCEC data. The precursor mass tolerance was set as 20 p.p.m and the MS/MS mass tolerance was set as 0.02 Da.

### RT and Fragment Ion Intensity Prediction

AutoRT (v2.0) (21, 33) was used for RT prediction, and pDeep3 (v1.0) (34) was used for fragment ion intensity prediction for each peptide sequence. Fine-tuning of the AutoRT model is recommended to account for dataset-specific variations in RT prediction. For pDeep3, if the MS/MS data in the new dataset are generated using similar mass spectrometers with similar settings to the pretrained models, fine-tuning may not be necessary as the pretrained models can be directly applied. In this study, we fine-tuned both models.

To construct experiment-specific datasets for fine-tuning, we included both phosphorylated and non-phosphorylated peptide identifications passing 1% FDR (estimate by PGA R package (v1.15.1) (35)) filtering at both PSM and peptide levels, and the phosphorylated peptide identifications were also required to have higher than 0.75 phosphoRS (v3.1) (16) site localization probability. For the test data, we randomly selected 500 identifications from the experiment-specific datasets, while the remaining identifications were used for the training datasets. The AutoRT algorithm automatically splits the training datasets into two parts: the training data for model training and the validation data for monitoring the model’s performance during training and deciding when to apply early stopping to prevent overfitting. Since the pDeep3 algorithm allows only a maximum of 2 epochs, early stopping is not necessary and thus all the training datasets were used for model training.

For RT prediction, we utilized the “phosphorylation_sty” model as the base model from the “ptm_base_model” folder in AutoRT, which is available at https://github.com/bzhanglab/AutoRT. The base model was fine-tuned using the experiment-specific data for a maximum of 40 epochs, with a batch size of 64. We implemented early stopping based on the validation loss, allowing for a maximum of 10 epochs with no improvement. For fragment ion intensity prediction, we fine-tuned the phosphorylation-specific base model of pDeep3, which is available at https://github.com/pFindStudio/pDeep3, using transfer learning with the experiment-specific data for a maximum of 2 epochs, with a batch size of 1024 to create experiment-specific models. The parameters and training models for all datasets have been uploaded to Zenodo (https://zenodo.org/records/10049730).

The experiment-specific models were then used to predict RT and MS/MS spectrum for all peptide isoforms of all identified peptides.

### Deep Learning-Facilitated Site Localization

For each phosphorylated site candidate i in an identified peptide sequence corresponding to spectrum S, two deep learning-derived scores were used to adjust the phophosRS localization score of this candidate site i (${PhosphoRSScore}_{i}$).

The first deep learning-derived score is the spectrum similarity (SS) between a predicted MS/MS spectrum $S_{p}$ and corresponding experimental MS/MS spectrum $S_{e}$. Several MS/MS spectrum similarity calculation methods were investigated, including dot product (DP), square root dot product (srDP), spectral contrast angle (SA), Pearson correlation coefficient (PCC), unweighted entropy distance (unwEntropy) (36), and entropy distance (Entropy) (36). Let $I_{p}$ and $I_{e}$ be two vectors containing the peak intensities from $S_{p}$ and $S_{e}$, respectively; similarity between $S_{p}$ and $S_{e}$ can be calculated by the six different scoring methods as follows:

$$DP=\frac{\sum I_{p}\times I_{e}}{\sqrt{\sum {I_{p}}^{2}}\times \sqrt{\sum {I_{e}}^{2}}}$$

$$srDP=\frac{\sum \sqrt{I_{p}}\times \sqrt{I_{e}}}{\sqrt{\sum I_{p}}\times \sqrt{\sum I_{e}}}$$

$$SA=1-\frac{2\;\cos^{-1}\left(I_{p}\times I_{e}\right)}{\pi }$$

$$PCC=\frac{\sum \left[\left(I_{p}-{\bar{I}}_{p}\right)\left(I_{e}-{\bar{I}}_{e}\right)\right]}{\sqrt{\sum {\left(I_{p}-{\bar{I}}_{p}\right)}^{2}\sum {\left(I_{e}-{\bar{I}}_{e}\right)}^{2}}}$$

$$unwEntropy=-\frac{2\times S_{pe}-S_{p}-S_{e}}{\ln\left(4\right)},S_{I}=\sum_{i}I_{i}\;\ln\left(I_{i}\right)$$

$$Entropy=-\frac{2\times S_{pe}^{'}-S_{p}^{'}-S_{e}^{'}}{\ln\left(4\right)},S_{I}^{'}=\sum_{i}I_{i}^{'}\;\ln\left(I_{i}^{'}\right),I^{'}=I^{w},w=0.25+S\times 0.5\;\left(S<1.5\right)$$

DP, PCC, unwEntropy, and Entropy were calculated using the Spectral Entropy Python package (36). srDP and SA were calculated by python scripts following the equations.

The second deep learning-derived score is the retention time difference between predicted RT (${RT}_{p}$) of the identified peptide sequence with phosphorylated site candidate *i* and experimentally observed RT (${RT}_{e}$) of the corresponding spectrum. Two methods were used to calculate the difference between ${RT}_{p}$ and ${RT}_{e}$. The first is delta RT (DRT):

$$DRT=\left|{RT}_{p}-{RT}_{e}\right|$$

The closer DRT is to 0, the closer ${RT}_{p}$ is to ${RT}_{e}$. The second is RT ratio (RTR):

$$RTR=\frac{\min\left({RT}_{p},{RT}_{e}\right)}{\max\left({RT}_{p},{RT}_{e}\right)}$$

The closer RTR is to 1, the closer ${RT}_{p}$ is to ${RT}_{e}$.

For a candidate site i of an identified peptide sequence corresponding to spectrum S, the following equations were used to adjust its PhosphoRS localization score by integrating SS score and DRT score or RTR score:

$${Score}_{i}={PhosphoRSScore}_{i}\times \frac{{SS}_{i}}{\max_{i}\left({SS}_{i}\right)}\times \frac{\min_{i}\left({DRT}_{i}\right)}{{DRT}_{i}}$$

or:

$${Score}_{i}={PhosphoRSScore}_{i}\times \frac{{SS}_{i}}{\max_{i}\left({SS}_{i}\right)}\times \frac{{RTR}_{i}}{\max_{i}\left({RTR}_{i}\right)}$$

where $\max_{i}\left({SS}_{i}\right)$ and $\max_{i}\left({RTR}_{i}\right)$ are the maximum SS and RTR for all the phosphorylated site candidates in the identified peptide sequence, respectively, and $\min_{i}\left({DRT}_{i}\right)$ is the minimum for all the phosphorylated site candidates in the identified peptide sequence.

Finally, the site localization probability of phosphorylated site candidate i was calculated by transforming the localization score through the base 10 SoftMax transformation formula as previously described (28):

$${Probability}_{i}=\frac{10^{\frac{{Score}_{i}}{10}}}{\sum_{i}10^{\frac{{Score}_{i}}{10}}}$$

### Deep Learning–Facilitated PSM Rescoring

The localization probabilities for all potential sites are predicted and stored for each spectrum. During rescoring, the stored probabilities are used for the selection of the best phosphorylation site candidate with the largest site localization probability as the site identification of spectrum S. Following the DeepRescore strategy (23), three feature sets (Supplemental Table S2) were extracted from the original PSM identifications or calculated based on the selected phosphorylated site and non-phosphorylated identifications. The first set comprised search engine-specific features, the second set comprised search engine-independent features, and the last set comprised deep learning-derived features, including both RTR and SS computed based on the entropy distance. Using the semi-supervised support vector machine (SVM)-based Percolator rescoring model (v3.4) (37), all the above features were integrated to compute a new score for each PSM.

### False Localization Rate (FLR) Calculation for the Synthetic Dataset

For each method, all phosphorylated PSMs with correctly identified sequence were sorted by the site localization probability in descending order, and the FLR was calculated as:

$$FLR=\frac{FL}{TL+FL}$$

where TL and FL are the numbers of true and false localization sites at the site localization probability cutoff.

### Phosphosite Level Quantification

The single site-level quantitation procedure was written in Python 3 (Python Software Foundation. Python Language Reference http://www.python.org), along with third-party libraries NumPy (v1.19.5) (38) and Pandas (v1.3.4, mckinney-proc-scipy-2010, reback2020pandas).

First, any identical modified peptide sequences (different PSMs; different scans with the same modified amino acid sequence) are combined into one record. Quantities are summed, and the best database search score is kept, creating a table unique on the modified sequence. Next, the table is expanded to produce a separate record for each modification (*e.g.*, a doubly phosphorylated modified sequence is duplicated into two records, one specific to each position). From here, a 15-mer site identifier is calculated for each record. Final site level quantitation is produced by summing each group of 15mers. This procedure is performed independently for each entry in the reference protein database. Phosphosite abundances were log2 transformed and median normalized for downstream analyses.

### Kinase Activity Inference

Kinase activity scores were inferred for each data table using the Kinase-Substrate Enrichment Analysis (KSEA) algorithm (39) implemented in R. Kinase and kinase family target phosphorylation sites collected from several sources and used to evaluate the GPS 5.0 target prediction model were used for the inference here (Supplemental Table S4 from Wang *et al.* (40)). For this study, we required measurements for at least five kinase targets in a given sample for the HCC dataset for kinase activity inference.

### Liver Cancer Organoid Culture and Afatinib Response Testing

Liver cancer specimens were collected from patients who underwent surgical resection at Zhongshan Hospital of Fudan University with signed informed consent forms. Liver cancer tissues were minced and digested at 37 °C in phosphate buffer saline (PBS, Gibco) containing Collagenase Type IV (5 mg/ml, Gibco) for 30 to 60 min with gentle shaking. After digestion, the suspension was filtered through a 100-mm cell strainer and centrifuged consecutively at 1000 rpm, 800 rpm, and 600 rpm for 5 min, respectively. The pellet was resuspended in a cold organoid culture medium and then mixed 1:2 with Matrigel (Corning) to reach a density of 4000 cells per 50 ml before seeding into a 24-well culture plate. After Matrigel solidification at 37 °C, an organoid culture medium was added to each well and organoids were cultured in a humidified incubator at 37 °C with 5% CO_2_.

For Afatinib (Selleck) response testing, organoids were gently digested and seeded into 384-well plates (Corning) at a density of 500 cells per well in a volume of 15 ml (1:1 mixture of culture medium and Matrigel) by Multidrop Combi Reagent Dispenser (Thermo Fisher Scientific). After incubation at 37 °C to make the Matrigel-medium mixture solidify, 35 ml of the pre-warmed culture medium was added into each well. After 72-h incubation, organoids were treated with serial dilutions of Afatinib using a D300e Digital Dispenser (Tecan). After another 72-h drug treatment, 20 ml of CellTiter Glo 3D (Promega) was added to each well followed by measuring the luminescent signals using an EnVision Multilabel Reader (PerkinElm) to determine the cell viability. Three replicative wells were measured. AUC was calculated with the equation:

$$AUC=\frac{\sum_{i=1}^{n}\left(V_{i}-V_{i-1}\right)\times \left(\mathrm{Log}\;C_{i}-\mathrm{Log}\;C_{i-1}\right)}{2\times \left(\mathrm{Log}\;C_{\max}-\mathrm{Log}\;C_{\min}\right)}$$

where $C_{i}$ stands for each concentration and $V_{i}$ stands for the relative cell viability at $C_{i}$. $C_{\max}$ and $C_{\min}$ were the maximum and minimum concentration for each drug in our screening.

### Western Blot Analysis of EGFR_Y1068

For the analysis of phospho-EGFR inhibition, 5 μM afatinib was added into the culture medium, and organoids were collected after incubation for 2 h. The organoid samples were collected and lysed in RIPA buffer supplemented with protease and phosphatase inhibitor cocktail (Beyotime). The samples were quantified by BCA Protein Assay Kit (Beyotime), separated by 10% SDS-PAGE (Beyotime), and transferred to a PVDF membrane (Millipore). The membrane was blocked with QuickBlock Western (Beyotime) and incubated with primary antibody (Phospho-EGF Receptor (Tyr1068) (D7A5), Cell Signaling Technology, CST-3777, 1:1000 dilution) at 4 °C overnight followed by secondary antibody incubation. The protein bands were visualized using ChemistarTM ECL Western Blotting Substrate (Tanon).

## Results

### DeepRescore2 Workflow

**Fig. 1:**
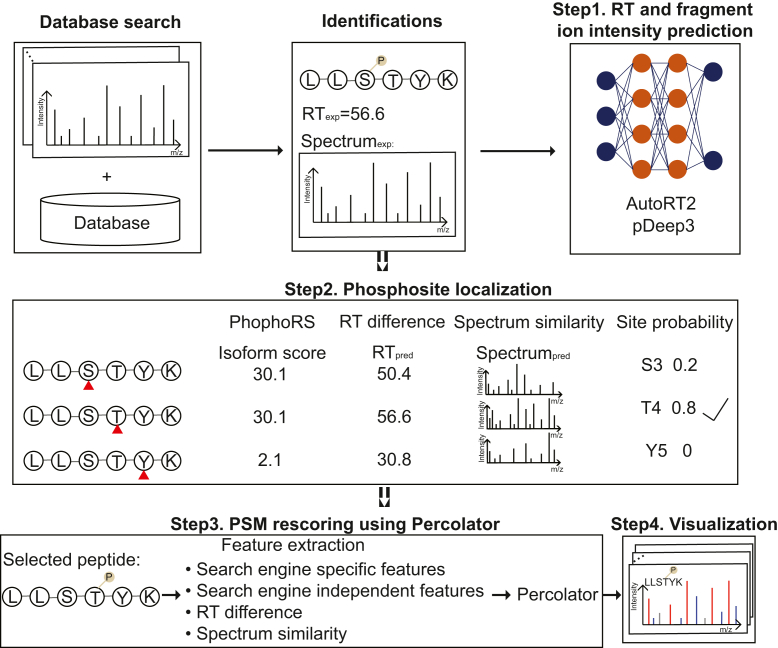
**DeepRescore2 workflow for phosphopeptide identification and phosphosite localization.** After searching the MS/MS spectra against a protein database using a search engine, DeepRescore2 processes the search results in four steps: (1) RT and fragment ion intensity prediction for all identified peptide sequences with all possible phosphosite localizations using deep learning models fine-tuned with confidently identified PSMs from the database search. (2) Phosphosite localization by combining site localization score from PhosphoRS and deep learning prediction derived spectrum similarity and retention time (RT) difference scores. (3) PSM rescoring using Percolator based on search engine specific features, search engine independent features, and spectrum similarity and retention time difference scores. (4) Visualization of the PSMs by PDV.

Figure 1 depicts the overall DeepRescore2 workflow, which processes the results of database searching in four steps to improve phosphopeptide identification and phosphosite localization (Experimental Procedures). First, based on the confidently identified PSMs from database searching, RT and fragment ion intensity prediction models are trained using AutoRT (21, 33) and pDeep3 (34), respectively, and then used to predict RTs and MS/MS spectra for all identified peptide sequences with all possible phosphosite localizations (*i.e.*, peptide isoforms). Second, for each peptide isoform, a probability score is computed taking into consideration the PhosphoRS score (16), RT difference between predicted and experimentally observed RTs, and spectrum similarity between predicted and experimentally observed spectra, and then phosphosite localization is determined based on the combined probability score. Third, PSM rescoring is performed using the semi-supervised Percolator algorithm (37), which integrates search engine-specific features, search engine-independent features, and the two deep learning-derived features to improve the accuracy and sensitivity of phosphopeptide identification. Finally, identified PSMs can be manually validated using the visualization tool PDV (41).

### Evaluation of RT and Fragment Ion Intensity Predictions

The DeepRescore2 workflow relies on accurate predictions of RT and fragment ion intensity. These predictions are achieved by utilizing AutoRT for RT predictions and pDeep3 for fragment ion intensity predictions. To improve the performance of these models, experiment-specific data was used to fine-tune their pre-trained base models (Experimental Procedures). To assess the potential overfitting of the fine-tuned deep learning models, we evaluated their performance on the train, validation, and test PSMs in a label-free dataset from human osteosarcoma cell line U2OS (30), and a TMT dataset on UCEC from the CPTAC (31). To save computational resources, the method evaluation was based on three raw files from the U2OS study and one TMT plex from the UCEC study (Supplemental Table S1). Both datasets were searched using four search engines Comet, MaxQuant, MS-GF+, and X!Tandem, respectively (Experimental Procedures). Only identifications within the 1% FDR limit at both PSM and phosphopeptide levels and a site localization probability greater than 0.75 were considered confident identifications.

The predicted RT values showed excellent alignment with the observed RT values in both the training and validation datasets, with a Spearman correlation close to 1 (Supplemental Fig. S2, *A* and *B*). Although the performance on the test dataset was slightly lower, the Spearman correlation remained around 0.9 across all four search engines for both the label-free and TMT datasets. In evaluating the similarities between the predicted and observed fragment ion intensities in both the training and test datasets, we found no significant differences for any of the four search engines in both the label-free and TMT datasets (Supplemental Fig. S2, *C* and *D*). These results indicate that the models were able to accurately predict RT and fragment ion intensity and generalize well to the test datasets.

### Benchmarking of Deep Learning-Facilitated Phosphosite Localization

**Fig. 2:**
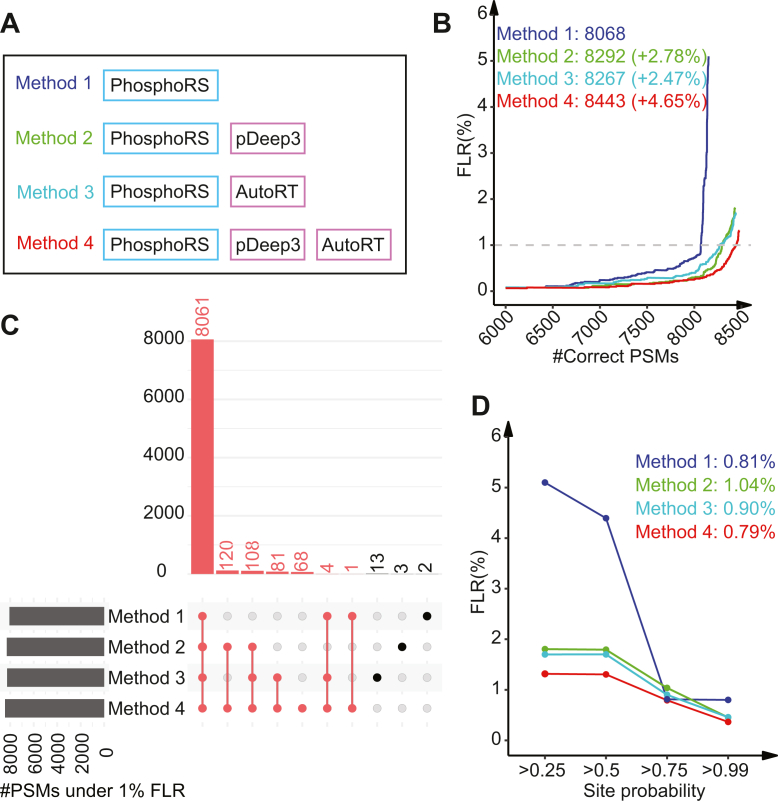
**Benchmarking of deep learning-facilitated phosphosite localization on the synthetic dataset.***A*, four site localization methods were benchmarked. *B*, the number of correctly localized PSMs at different levels of PSM FLR are shown for the four methods, respectively. The numbers of correctly localized PSMs at 1% FLR and the percent increase compared with Method 1 (phosphoRS) are indicated. *C*, upSet plot comparing the PSM identifications from the four methods. The number of correctly localized PSMs by Method 4 are marked as red. *D*, the FLRs at different site probability cutoffs are shown. The FLRs at 0.75 site probability are indicated.

We benchmarked the performance of four site localization methods, PhosphoRS alone (Method 1) and its combination with either spectrum similarity (Method 2), RT difference (Method 3) or both (Method 4), on MaxQuant search results from a synthetic phosphopeptide dataset (29), in which the ground truth is known and the FLR can be determined precisely (Fig. 2*A*, Supplemental Table S1). Of note, no rescoring was involved in this section. Spectrum similarity was computed based on Entropy (36), which outperformed the other five spectrum similarity computation methods including DP, srDP, SA, PCC, and unwEntropy in our comparative analysis (Experimental Procedures, Supplemental Fig. S3*A*). RT difference was computed based on RTR, which outperformed the alternative method DRT in our comparative analysis (Experimental Procedures, Supplemental Fig. S3*B*).

Among the four site localization methods, the ones incorporating deep learning-derived features consistently outperformed the PhosphoRS alone method at different levels of FLRs, with the best performance observed for the method simultaneously incorporating both features (Fig. 2*B*). At 1% FLR, compared with Method 1, Methods 2, 3, and 4 increased the number of correctly identified PSMs by 2.78%, 2.47%, and 4.65%, respectively. Almost all PSMs correctly identified by Method 1, 2, or 3 were also correctly identified by Method 4 (Fig. 2*C*). To illustrate the complementary contributions of RT prediction and fragment ion intensity prediction to the superior performance of Method 4, we investigated the RT difference and spectrum similarity scores for two sets of PSM identifications. The first set consisted of 81 PSMs successfully identified by Methods 3 and 4 but not Methods 1 and 2, while the second set consisted of 120 PSMs successfully identified by Methods 2 and 4 but not Methods 1 and 3 (Fig. 2*C*). In a majority of these cases, the experimental spectra lacked the ions necessary to precisely determine the phosphosite localization, resulting in a localization probability close to 0.5 when using Method 1. Among the 81 cases, the incorporation of RT prediction helped distinguish the isoform with correct phosphosite localization from the putative isoforms (Supplemental Fig. S4*A*), whereas the incorporation of spectrum prediction did not show the same effect (Supplemental Fig. S4*B*). Consequently, Method 3, but not Method 2, produced high phosphosite localization probabilities for this set of PSMs (Supplemental Fig. S4*C*). On the other hand, for the 120 PSMs, opposite trends were observed (Supplemental Fig. S4, *D*–*F*). These results suggest that the integration of the complementary information sources in Method 4 leads to improved performance.

We further investigated the relationship between computed site localization probabilities and the true FLR. Among the four methods, Method 4 was associated with the lowest FLRs for all four site localization probability cutoffs investigated, with an FLR of 0.79% observed for the cutoff of 0.75 (Fig. 2*D*). Moreover, with the site probability cutoff of 0.75, all other methods were also able to achieve an FLR of around 1%, which is acceptable for typical phosphoproteomics studies.

### Benchmarking of Deep Learning-Facilitated Phosphopeptide Identification

**Fig. 3:**
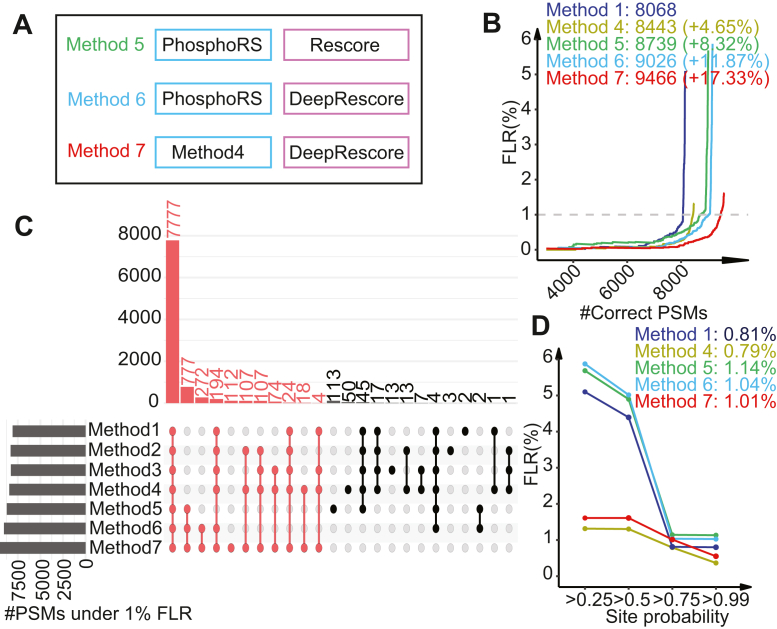
**Benchmarking of deep learning-facilitated phosphopeptide identification on the synthetic dataset.***A*, three phosphopeptide identification methods were benchmarked. *B*, the number of correctly localized PSMs at different levels of PSM FLR are shown for the three methods, respectively, together with Methods 1 and 4. The numbers of correctly localized PSMs at 1% FLR and the percent increase compared with Method 1 (phosphoRS) are indicated. *C*, upSet plot comparing the PSM identifications from all seven methods. The number of correctly localized PSMs by Method 7 is marked as red. *D*, the FLRs at different site probability cutoffs are shown. The FLRs at 0.75 site probability are indicated.

Based on MaxQuant search results from the synthetic phosphopeptide dataset, we further benchmarked three phosphopeptide identification methods with different combinations of the site localization and PSM rescoring algorithms, using Method 1 (PhosphoRS only) as the baseline method for comparison (Fig. 3*A*). The three methods included PhosphoRS followed by a traditional PSM rescoring algorithm without using deep learning-derived features (Method 5), PhosphoRS followed by our proposed DeepRescore algorithm for PSM rescoring (Method 6), and Method 4 followed by DeepRescore (Method 7). In DeepRescore, the two deep learning-derived features, spectrum similarity and RT difference, were computed based on entropy distance and RT ratio, respectively, based on their superior performance as compared to alternative methods (Experimental Procedures, Supplemental Fig. S5, *A* and *B*).

Method 6 and 7 consistently outperformed Method 1 and 5 at different levels of FLRs, and Method 5 outperformed Method 1, but only in the region where FLR was higher than 0.4% (Fig. 3*B*). At 1% FLR, compared with Method 1, Methods 5, 6, and 7 increased the number of correctly identified PSMs by 8.32%, 11.87%, and 17.33%, respectively. These increases were much higher than those observed for Methods 2, 3, and 4, suggesting a dominant role of PSM rescoring, especially DeepRescore, in the observed performance gain. The largest performance gain was from Method 7, which leverages deep learning-derived features in both site localization and PSM rescoring. Almost all PSMs correctly identified by the other six methods were also correctly identified by Method 7 (Fig. 3*C*). Similar to Methods 1 to 4, Methods 5 to 7 were also able to achieve an FLR of around 1% with the site probability cutoff of 0.75 (Fig. 3*D*).

The initial database search results consisted of both true and false identifications, and both were used as targets or positive samples during the training of AutoRT and pDeep3 models. In the synthetic dataset, the true and false identifications could be separated, allowing us to evaluate the potential impact of including false identifications as positive training samples on the final results. We compared the score distributions of true targets, false targets, and decoy identifications for Method 1 (without rescoring), Method 5 (rescoring without deep learning-derived features), and Method 7 (rescoring with deep learning-derived features). As expected, rescoring enhanced the score difference between true targets and decoys, irrespective of whether deep learning-derived features were used or not (Supplemental Fig. S6). At a 1% FDR, the ratio between the numbers of true and false targets for Method 5 was 9.17x, which was comparable to that of Method 1 (10.58x). Interestingly, Method 7 reported not only an increased number of true targets but also a reduced number of false targets, resulting in the highest true target to false target ratio (14.32x). These results highlight that incorporating deep learning-derived features in both the relocalization and rescoring process within the workflow effectively addresses false identifications in the initial database search results, ultimately enhancing sensitivity and accuracy.

Together, data from our benchmarking study on a synthetic dataset clearly revealed superior performance for Method 7 compared with other competing methods. Method 7, which leverages deep learning-based retention time and fragment ion intensity predictions in both phosphosite localization and PSM rescoring, was referred to as DeepRescore2 and used in all subsequent studies in this paper.

### Sensitivity Improvement in Real-World Biological Datasets

Next, we assessed the performance of DeepRescore2 in two real-world biological datasets, including the U2OS label-free dataset (30) and the UCEC TMT dataset (31) (Experimental Procedures, Supplemental Table S1). For each dataset, the search results from Comet, MaxQuant, MS-GF+, and X!Tandem, respectively, were processed using DeepRescore2 or PhosphoRS.

**Fig. 4:**
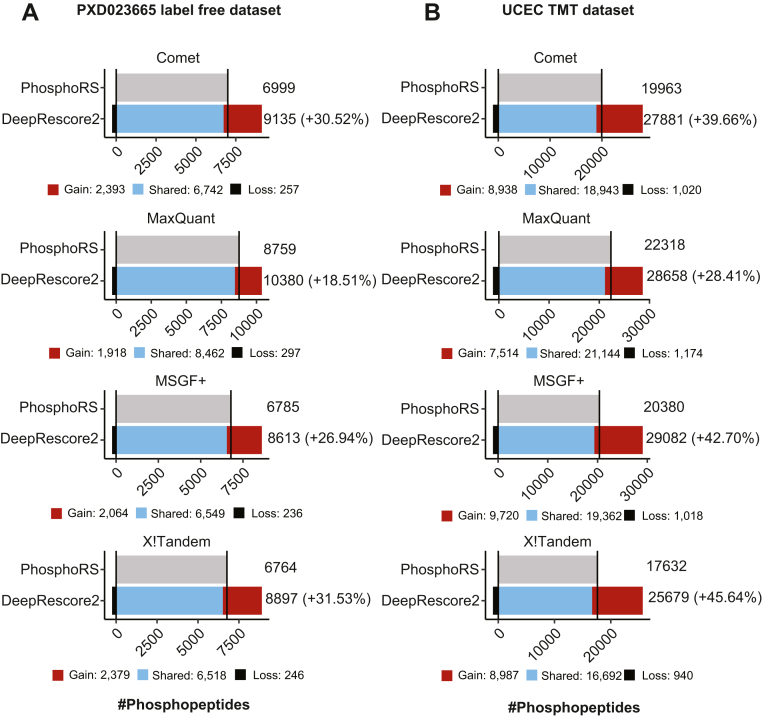
**Performance evaluation based on peptide identification in two biological datasets using different search engines in combination with PhosphoRS or DeepRescore2.***A*, the numbers of phosphopeptides identified from a label-free phosphoproteomic dataset, PXD023665, by four search engines in combination with phosphoRS or DeepRescore2, respectively. *B*, the numbers of phosphopeptides identified from the UCEC TMT phosphoproteomic dataset by four search engines in combination with phosphoRS or DeepRescore2, respectively. Gain: phosphopeptides identified by DeepRescore2 but not PhosphoRS. Shared: phosphopeptides identified by both DeepRescore2 and PhosphoRS. Loss: phosphopeptides identified by PhosphoRS but not DeepRescore2.

In the U2OS label-free dataset, when used in combination with DeepRescore2, Comet, MaxQuant, MS-GF+, and X!Tandem identified 9,135, 10,380, 8613, and 8897 phosphorylated peptides, respectively, which were 31%, 19%, 27%, and 32% higher than the numbers reported when they were used in combination with PhosphoRS (Fig. 4*A*, Supplemental Table S3). Furthermore, we investigated the consistency of phosphosite identification and localization along an Extracted Ion Chromatogram (XIC), using a 0.5 tolerance window around the RT and mass values. For all four search engines, DeepRescore2 exhibited higher stability along peptide elution and displayed less dependence on the absolute precursor intensity at the time of the MS/MS event compared to PhosphoRS (Supplemental Fig. S7).

In the UCEC TMT dataset, when used in combination with DeepRescore2, Comet, MaxQuant, MS-GF+, and X!Tandem identified 27,881, 28,658, 29,082, 25,679 phosphorylated peptides, respectively, which were 40%, 28%, 43%, 46% higher than the numbers reported when they were used in combination with PhosphoRS (Fig. 4*B*, Supplemental Table S3). In both the label-free and the TMT datasets, DeepRescore2 identifications covered almost all PhosphoRS identifications for all search engines (Fig. 4). All above phosphopeptide-level observations were similarly observed at the PSM level (Supplemental Fig. S8, Supplemental Table S3). Together, these results clearly demonstrate that DeepRescore2 increases the sensitivity of phosphopeptide identification in real-world biological datasets.

### Increased Identification of Liver Cancer-Associated Phosphosites

Improved phosphopeptide identification is expected to enhance biological discoveries. To demonstrate this potential, we applied DeepRescore2 to a previously published HCC study (32), which included TMT-based phosphoproteomic analysis of paired treatment-naive tumors and normal adjacent tissues (NATs) from 159 HCC patients (Supplemental Table S1). The dataset was searched using MaxQuant (Experimental Procedures) and then processed using either PhosphoRS or DeepRescore2. Identifications within the 1% FDR limit at both PSM and phosphopeptide levels and a site localization probability greater than 0.75 were considered as confident identifications. Phosphosite level quantification was performed for confident identifications from PhosphoRS and DeepRescore2 (Experimental Procedures).

**Fig. 5:**
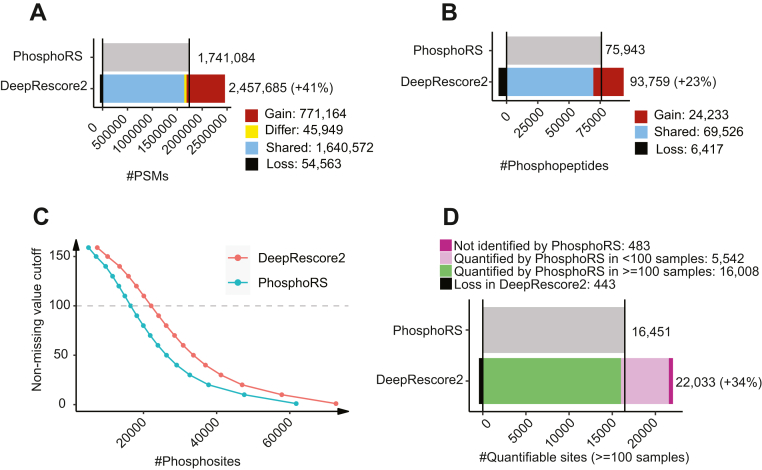
**Performance evaluation on a large liver cancer dataset.***A* and *B*, the numbers PSMs (*A*) and phosphopeptides (*B*) identified by MaxQuant in combination with phosphoRS or DeepRescore2, respectively. Gain: identified by DeepRescore2 but not PhosphoRS. Shared: identified by both DeepRescore2 and PhosphoRS. Loss: identified by PhosphoRS but not DeepRescore2. Differ: different sites identified by DeepRescore2 and PhosphoRS, respectively. *C*, the numbers of “quantifiable” phosphosites based on different levels of non-missing value cutoff, *i.e.*, numbers of samples with a non-missing value in tumors and NATs, respectively. *D*, the numbers of quantifiable phosphosites (*i.e.*, quantified in at least 100 tumor samples and 100 NAT samples) in the data tables generated by DeepRescore2 and PhosphoRS, respectively.

Across the whole cohort, DeepRescore2 identified 2,457,685 PSMs and 93,759 phosphopeptides, which increased the PhosphoRS-based PSM and phosphopeptide identifications by 41% and 23%, respectively (Fig. 5, *A* and *B*). Importantly, the vast majority of PSM and phosphopeptide identifications reported by PhosphoRS were covered by DeepRescore2 identifications. Phosphosite quantification matrices generated based on DeepRescore2 included more “quantifiable” phosphosites than those based on PhosphoRS identifications across a wide range of non-missing value cutoffs (Fig. 5*C*). For downstream analyses, we defined quantifiable sites as those quantified in at least 100 tumor samples and 100 NAT samples. With this definition, DeepRescore2 identified 22,033 quantifiable sites, which was 34% more than those identified by PhosphoRS (Fig. 5*D*, Supplemental Table S4). Only 443 out of the 16,451 (2.7%) quantifiable sites identified by PhosphoRS were not identified by DeepRescore2.

**Fig. 6:**
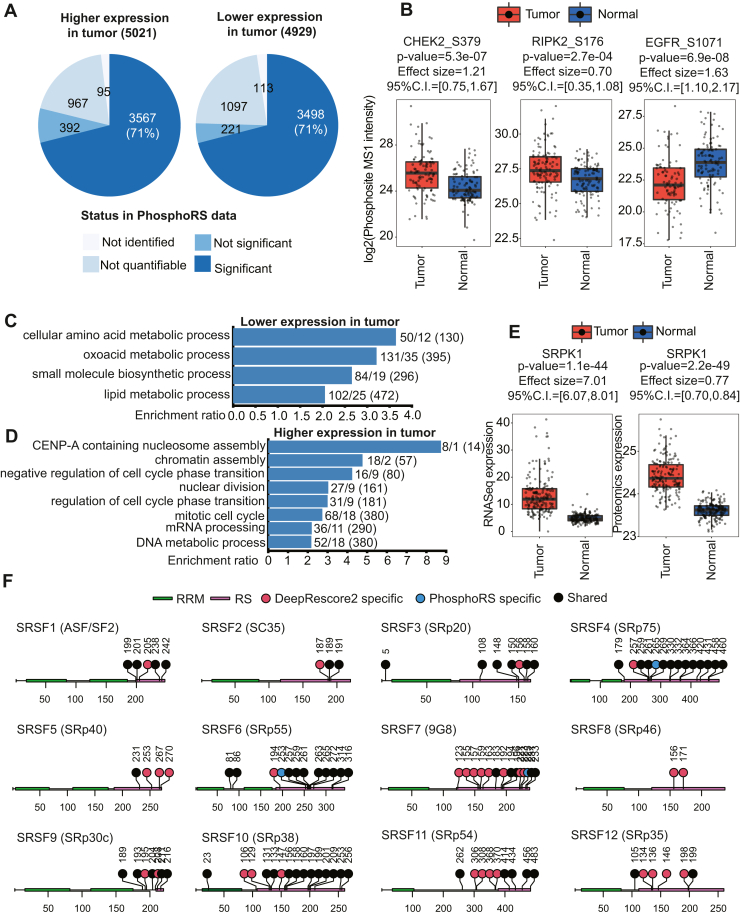
**Differentially expressed phosphosites between tumor and NAT samples in the liver cancer dataset.***A*, the numbers of significantly altered (FDR < 0.01) phosphosites identified in DeepRescore2-derived data and their status in the analysis of PhosphoRS-derived data. *B*, comparison of phosphosite abundance in tumors and NATs for CHEK2_S379, RIPK2_S176, and EGFR_S1071, respectively. *C*, gene ontology enrichment analysis results of the parent proteins of the 4929 significantly down-regulated phosphosites. *D*, gene ontology enrichment analysis results of the parent proteins of the 5021 significantly up-regulated phosphosites. In *C* and *D*, the numbers at the end of each bar represent the number of parent proteins identified by both PhosphoRS and DeepRescore2, the number of parent proteins identified by DeepRescore2 alone, and the total number of parent proteins in the pathway, respectively. *E*, comparison of SRPK1 abundance between tumors and NATs at mRNA and protein levels, respectively. *F*, differentially expressed phosphosites detected in DeepRescore2-or PhosphoRS-derived data on 12 SR proteins. *p* values are based on the Wilcoxon rank sum test.

To identify liver cancer-associated phosphosites, we assessed the differences in phosphosite abundance between tumors and paired NATs. Among the 22,033 quantifiable phosphorylation sites reported by DeepRescore2, 5021 were significantly increased and 4929 were significantly decreased in tumors compared to paired NATs (adj. *p* < 0.01, Wilcoxon signed-rank test). Of these, only 3567 (71%) and 3498 (71%) were also found to be significantly altered based on PhosphoRS-derived data, and others were not identified (95 + 113), not quantifiable (967 + 1097), or not significantly altered (392 + 221) (Fig. 6*A*, Supplemental Table S4). Phosphosites that showed statistically significant alterations and were not identified or quantifiable in the PhosphoRS-derived data included both known functional sites on established cancer genes such as CHEK2_S379 (known to induce CHEK2 enzymatic activity (42)) and EGFR_S1071 (associated with EGFR desensitization (43)), as well as sites on less explored genes such as RIPK2_S176 (known to induce RIPK2 enzymatic activity (44)) (Fig. 6*B*). These findings expanded the pool of putative liver cancer associated phosphosites for further experimental validation.

At the pathway level, parent genes of the 4929 decreased sites were enriched in different metabolic processes (Fig. 6*C*), consistent with the essential role of the liver in metabolism. Parent genes of the 5021 increased sites were enriched in biological processes related to CENP-A containing nucleosome assembly, chromatin assembly, negative regulation of cell cycle phase transition, nuclear division, regulation of cell cycle phase transition, mitotic cell cycle, mRNA processing, DNA metabolic process, etc (Fig. 6*D*). Protein phosphorylation plays an important role in many mRNA processing events, including pre-mRNA splicing (45). Phosphorylation status greatly modulates the activity of SR proteins, a family of nuclear RNA-binding proteins involved in the regulation of both constitutive and alternative splicing (46). In our tumor *versus* NAT comparison, 98 phosphosites from the 12 SR proteins were significantly increased in tumors. Moreover, 78 (80%) of the 98 phosphosites were on serines located in the RS domain. This region is specifically recognized by SRPK1 (47), the most well-studied SR protein kinase (48). In accordance with this observation, SRPK1 showed significantly increased mRNA and protein abundance in tumors compared with NATs in this cohort (Fig. 6*E*). Thus, data from both kinase and substrates support the role of SPRK1 activity in liver cancer development. Importantly, only 55 out of the 78 phosphosites (71%) in the RS domain were identified as significantly increased in the analysis of the PhosphoRS-derived dataset (Fig. 6*F*). Together, DeepRescore2 provides a more comprehensive catalog of liver cancer-associated phosphosites, linking functional phosphosites and biological processes to liver cancer oncogenesis, and expanding the list of putative substrates of liver cancer-associated SRPK1 activity.

### Increased Identification of Prognosis-Associated Phosphosites in Liver Cancer

**Fig. 7:**
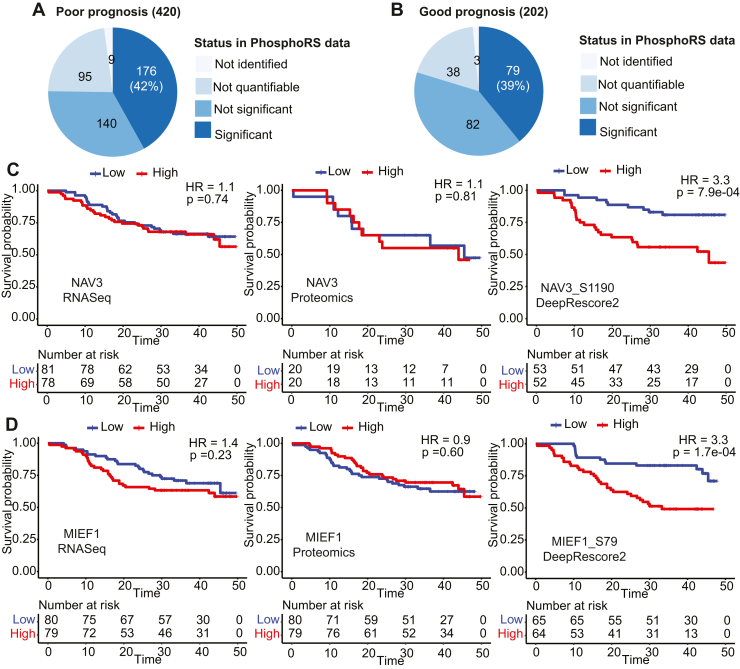
**Prognosis-associated phosphosites in the liver cancer dataset.***A*, the numbers of phosphosites significantly associated with poor prognosis (*p* < 0.01 and HR > 2) in DeepRescore2-derived data and their status in the analysis of PhosphoRS-derived data. *B*, the numbers of phosphosites significantly associated with good prognosis (*p* < 0.01 and HR < 0.5) in DeepRescore2-derived data and their status in the analysis of PhosphoRS-derived data. *C*, Kaplan Meier curves for patients stratified based on the median abundance of NAV3 RNA, NAV3 protein, and NAV3_S1190 phosphorylation, respectively. *D*, Kaplan Meier curves for patients stratified based on the median abundance of MIEF1 RNA, MIEF1 protein, and MIEF1_S79 phosphorylation, respectively. *p* values are based on the log-rank test.

To identify prognosis-associated phosphosites in liver cancer, we further performed survival analysis based on overall survival (OS) data of the 159 HCC patients. Among the 22,033 quantifiable phosphorylation sites reported by DeepRescore2, 420 were significantly associated with poor prognosis (*p* < 0.01, hazard ratio (HR) > 2, log-rank test) and 202 were significantly associated with good prognosis (*p* < 0.01, HR < 0.5). Of these, only 176 (42%) and 79 (39%) were also found to be significantly associated with prognosis based on PhosphoRS-derived data, and others were not identified (9 + 3), not quantifiable (95 + 38), or not significantly associated (140 + 82) (Fig. 7, *A* and *B*, Supplemental Table S4). Among the top 10 poor prognosis-associated phosphosites with the largest HRs, only seven showed significant association with OS in PhosphoRS-derived data (Table 1). Moreover, eight out of the 10 phosphosites had a larger hazard ratio than those computed based on cognate mRNA and protein measurements (Table 1). In particular, NAV3_S1190 and MIEF1_S79 were significantly associated with poor prognosis based on DeepRescore2-but not PhosphoRS-derived data, while their cognate mRNA and protein were not significantly associated with OS (Fig. 7, *C* and *D*), suggesting a specific contribution of phosphorylation to the observed survival associations.

**Table 1:** Survival data for the top 10 poor prognosis-associated phosphosites identified by DeepRescore2 and their parent genes based on RNA and protein measurements

No.	Site	Phosphoproteomics				RNA-seq		Proteomics	
		DeepRescore2		PhosphoRS		*p*-value	HR	*p*-value	HR
		*p*-value	HR	*p*-value	HR				
1	**DNAJB1_S16**	1.1e-07	4.9	1.3e-07	4.8	0.03	1.8	0.13	1.5
2	**DTL_S655**	1.3e-04	3.9	NA	NA	0.002	2.4	7.8e-04	4.0
3	**CEP170_T628**	7.6e-05	3.9	3.2e-03	2.7	0.06	1.6	2.0e-04	2.8
4	**PKM_S127**	2.8e-06	3.8	9.4e-06	3.5	5.3e-06	3.6	2.0e-03	2.3
5	**PSMD11_S298**	4.0e-05	3.7	1.8e-04	3.4	0.03	1.8	0.83	0.9
6	**ARHGEF1_S631**	1.1e-05	3.5	1.1e-04	3.0	0.58	1.2	0.25	1.4
7	**FXR1_S432**	4.6e-04	3.5	1.1e-03	3.1	0.11	1.5	0.03	1.8
8	**ANLN_S72**	9.4e-05	3.3	4.0e-04	3.0	8.5e-06	3.5	0.007	2.2
9	**NAV3_S1190**	7.9e-04	3.3	0.8	1.1	0.70	1.1	0.81	1.1
10	**MIEF1_S79**	1.7e-04	3.3	NA	NA	0.20	1.4	0.60	0.9

### EGFR Hyperactivation as a New Target in Poor-Prognosis Liver Cancer

Since kinases are important therapeutic targets, we next performed kinase activity inference for the 159 HCC tumor samples based on DeepRescore2-and PhosphoRS-derived phosphoproteomics datasets, respectively (Experimental Procedures). Kinase activity was quantified for 134 and 120 kinases/kinase families based on DeepRescore2-and PhosphoRS-derived data, respectively. To identify prognosis-associated kinases, we performed survival analysis using kinase activity scores derived from DeepRescore2 and PhosphoRS data, respectively.

**Fig. 8:**
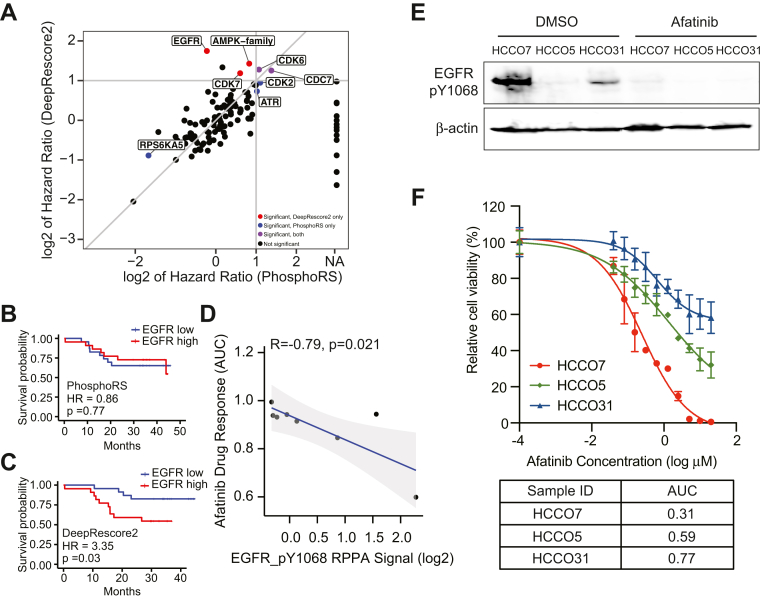
**Kinase activity analysis identifies EGFR hyperactivation as a new target in poor-prognosis liver cancer.***A*, scatterplot comparing hazard ratios of kinase activities inferred based on DeepRescore2-and PhosphoRS-derived data, respectively. *B*, Kaplan Meier curves for patients stratified based on median abundance of EGFR activity inferred based on PhosphoRS-derived data. *C*, Kaplan Meier curves for patients stratified based on median abundance of EGFR activity inferred based on DeepRescore2-derived data. *D*, correlation between afatinib response quantified based on AUC and EGFR_Y1068 abundance measured by RPPA. *E*, Western blot analysis of EGFR_Y1068 on three HCC organoids, HCCO7, HCCO5, and HCCO31, treated with DMSO or 5 μM afatinib for 2 h. *F*, relative cell viability as a function of afatinib concentration in the three organoids. Data were from three replicates.

The activities of eight kinases (EGFR, CDK7, AMPK-family, CDK6, CDC7, CDK2, ATR, and RPS6KA5) were significantly associated with OS (*p* < 0.05, HR > 2 or <0.5, log rank test) in one or both analyses (Supplemental Table S5), and analysis results based on the two datasets were comparable for the vast majority of kinases (Fig. 8*A*). One notable outlier was EGFR, for which higher inferred activity was associated with shorter OS, but only when the inference was made based on DeepRescore2 data (Fig. 8, *A*–*C*).

To examine whether EGFR hyperactivation could serve as a target for anti-cancer interventions in HCC, liver cancer cell line data from DepMap was used to correlate Reverse Phase Protein Array (RPPA) measurement of EGFR_Y1068, a site that is crucial to EGF-induced Ras/MAPK signaling (49), with response to afatinib, an EGFR-specific tyrosine kinase inhibitor. We observed a strong negative correlation between EGFR_Y1068 abundance and afatinib response as quantified by the area under the curve (AUC, smaller AUC corresponds to higher sensitivity) across the eight liver cancer cell lines (R=−0.79,p=0.021, spearman correlation, Fig. 8*D*), suggesting that EGFR_Y1068 levels could be a potential marker of response to EGFR inhibition.

To further confirm this finding from a cell line-based high-throughput study, we identified three HCC organoids with varied levels of EGFR_Y1068 phosphorylation by Western blot (Experimental Procedures). HCCO7 expressed a high level of EGFR_Y1068, whereas HCCO31 and HCCO5 had much lower levels of EGFR_Y1068 abundance (Fig. 8*E*). Upon afatinib treatment, a detectable decrease of EGFR_Y1068 was observed in all models, confirming drug target engagement (Fig. 8*E*). HCCO7 demonstrated high sensitivity to afatinib (AUC = 0.31, Experimental Procedures), while the other organoids were less sensitive (Fig. 8*F*). These results corroborate the results from the DepMap cell line analysis and support the use of EGFR_Y1068 as a marker of response to EGFR inhibitors in liver cancer. Together, our data demonstrate the power of improved phosphosite identification in improving kinase activity inference, leading to the identification of poor-prognosis-associated hyperactivated kinases as new targets for drug repurposing or development.

## Discussion

We developed DeepRescore2, a computational workflow that leverages deep learning prediction to improve phosphopeptide identification and phosphosite localization in phosphoproteomics data analysis. DeepRescore2 substantially increases the sensitivity of phosphopeptide identification in both synthetic and real-world biological datasets, and improved identification leads to an in-depth understanding of kinase signaling and deeper biological discoveries.

A key strength of DeepRescore2 is the integrative workflow that leverages complementary computational techniques including PhosphoRS scoring, semi-supervised machine learning through Percolator, and deep learning-based prediction of phosphopeptide RT and fragment ion intensities. Based on a synthetic dataset with known ground truth, our benchmarking study clearly showed that the combination of all these techniques collectively contributed to the ultimate best performance. The performance gain was observed when incorporating deep learning prediction in either phosphosite localization or PSM rescoring, but the latter provided a stronger sensitivity increase.

During the benchmarking process, we also comprehensively assessed the impact of different methods for computing spectrum similarity and RT difference on the performance of site localization and PSM rescoring (Supplemental Figs. S3 and S5). Our analyses revealed a substantial impact of method selection on the performance of both tasks. For spectrum similarity computation, entropy distance, a similarity scoring method recently proposed for metabolomics data analysis^32^, showed the best performance in our evaluation. For RT difference computation, a new method, RT ratio, outperformed the routinely used delta RT method. Although not the focus of this study, these results should be of particular interest to the proteomics and mass spectrometry community.

Applying DeepRescore2 to three real-world biological datasets demonstrated its application to both label-free and TMT phosphoproteomics datasets, and its flexibility to be combined with different search engines. Importantly, improved sensitivity in phosphopeptide identification directly translated into increased biological discoveries. Most of the phosphosites significantly associated with liver cancer development and prognosis have very limited information in the literature, opening new opportunities for further investigation. Increased phosphosite identification also led to improved kinase activity inference. One important finding is that EGFR hyperactivation is not only associated with poor prognosis in liver cancer but also associated with increased sensitivity to afatinib in both cell lines and organoids. Afatinib is approved for non-small cell lung cancer harboring EGFR mutations. It is also being investigated as a tissue-agnostic drug in a biomarker-guided phase 2 basket clinical trial where patients with tumors, regardless of location, harboring EGFR activating mutations are matched to treatment with afatinib (NCT02465060). Although EGFR inhibitors have not been approved for liver cancer, our finding may accelerate mechanism-based drug repurposing by incorporating EGFR_Y1068 as a patient stratification marker for clinical investigation.

One limitation of our study is that we performed the benchmarking analysis on only a subset of mass spectrometry runs from the synthetic dataset. This was done to allow us to compare seven different methods in a reasonable time frame. However, using the complete dataset could lead to more robust benchmarking results. Another limitation of the study is that our new method was combined only with the phosphosite localization algorithm phosphoRS. We chose phosphoRS as the baseline method because it showed relatively higher sensitivity compared with other phosphosite localization algorithms in previous evaluation studies (28, 29, 50). During our manuscript preparation, a new phosphosite localization algorithm, AScorePro, has been published and showed superior performance compared with its predecessor AScore (51). Because the deep learning-derived features are largely independent of the information used in AscorePro, we expect that combining deep learning-derived features with AscorePro could also improve phosphosite localization. However, this will need to be formally tested in the future. It would also be interesting to leverage the new Iterative Synthetically Phosphorylated Isomers (iSPI) resource (51) in future benchmarking efforts. Moreover, DeepRescore2 could be further improved by integrating additional deep learning-derived features, such as phosphosite probability prediction (21). However, such a method might introduce a bias into scoring towards previously identified phosphopeptides and thus additional benchmarking is required to thoroughly evaluate the impact and effectiveness of this approach. With the increasing use of other PTM profiling in biomedical research, DeepRescore2 can also be expanded to support other PTMs by incorporating deep learning-derived features trained on data of other PTMs, which will further broaden the impact of the DeepRescore2 workflow.

## Data Availability

Phosphoproteomics data used in this study were downloaded from public data repositories as described in the Experimental Procedures section. Search engine configuration files, databases used in this study, annotated spectra, and other result files are available at Zenodo (https://zenodo.org/records/10049730). Source code of DeepRescore2 is freely available at GitHub (https://github.com/bzhanglab/DeepRescore2), and it is distributed under the GNU General Public License v3.0.

## Supplemental data

This article contains supplemental data (4, 5, 6, 7, 16, 33, 34, 35, 37, 39).

## Conflict of interest

The authors declare the following financial interests/personal relationships which may be considered as potential competing interests: B. Z. received consulting fee from AstraZeneca. The remaining author declares no competing interests.
