# Supplementary material for: Deep Learning Prediction Boosts Phosphoproteomics-Based Discoveries Through Improved Phosphopeptide Identification
Source: Mol Cell Proteomics. 2023 Dec 26;23(2):100707. doi: 10.1016/j.mcpro.2023.100707 (PMC10831110; doi:10.1016/j.mcpro.2023.100707)
Supplement: Supplemental Data [file mmc1.pdf]

Deep learning prediction boosts phosphoproteomics-based discoveries through improved phosphopeptide identification

Xinpei Yi[1,2,5], Bo Wen[1,2], Shuyi Ji[3], Alexander B. Saltzman[4], Eric J. Jaehnig[1,2], Jonathan T. Lei[1,2], Qiang Gao[3], Bing Zhang[1,2*]

[1]*Lester and Sue Smith Breast Center, Baylor College of Medicine, Houston, TX 77030, USA*

[2]*Department of Molecular and Human Genetics, Baylor College of Medicine, Houston, TX 77030, USA*

[3]*Department of Liver Surgery and Transplantation, Liver Cancer Institute, Zhongshan Hospital and Key Laboratory of Carcinogenesis and Cancer Invasion of the Ministry of China, Fudan University, 180 Fenglin Road, Shanghai 200032, China.*

[4]*Mass Spectrometry Proteomics Core, Advanced Technology Cores, Baylor College of Medicine, Houston, TX 77030, USA*

[5]*Present address: Department of Bioinformatics and Biostatistics, School of Life Sciences and Biotechnology, Shanghai Jiao Tong University, Shanghai 200240, China.*

*Correspondence: bing.zhang@bcm.edu (B.Z.)

Supplemental Figures

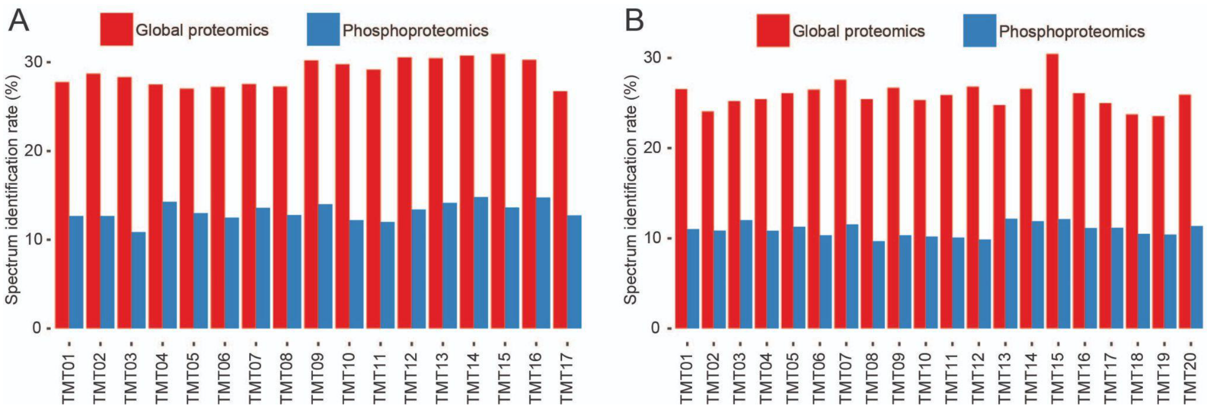

Figure S1: Spectrum identification rate comparison between global proteomics and phosphoproteomics. (A) CPTAC UCEC datasets analyzed by the common data analysis pipeline (CDAP). (B) CPTAC HNSCC datasets analyzed by CDAP.

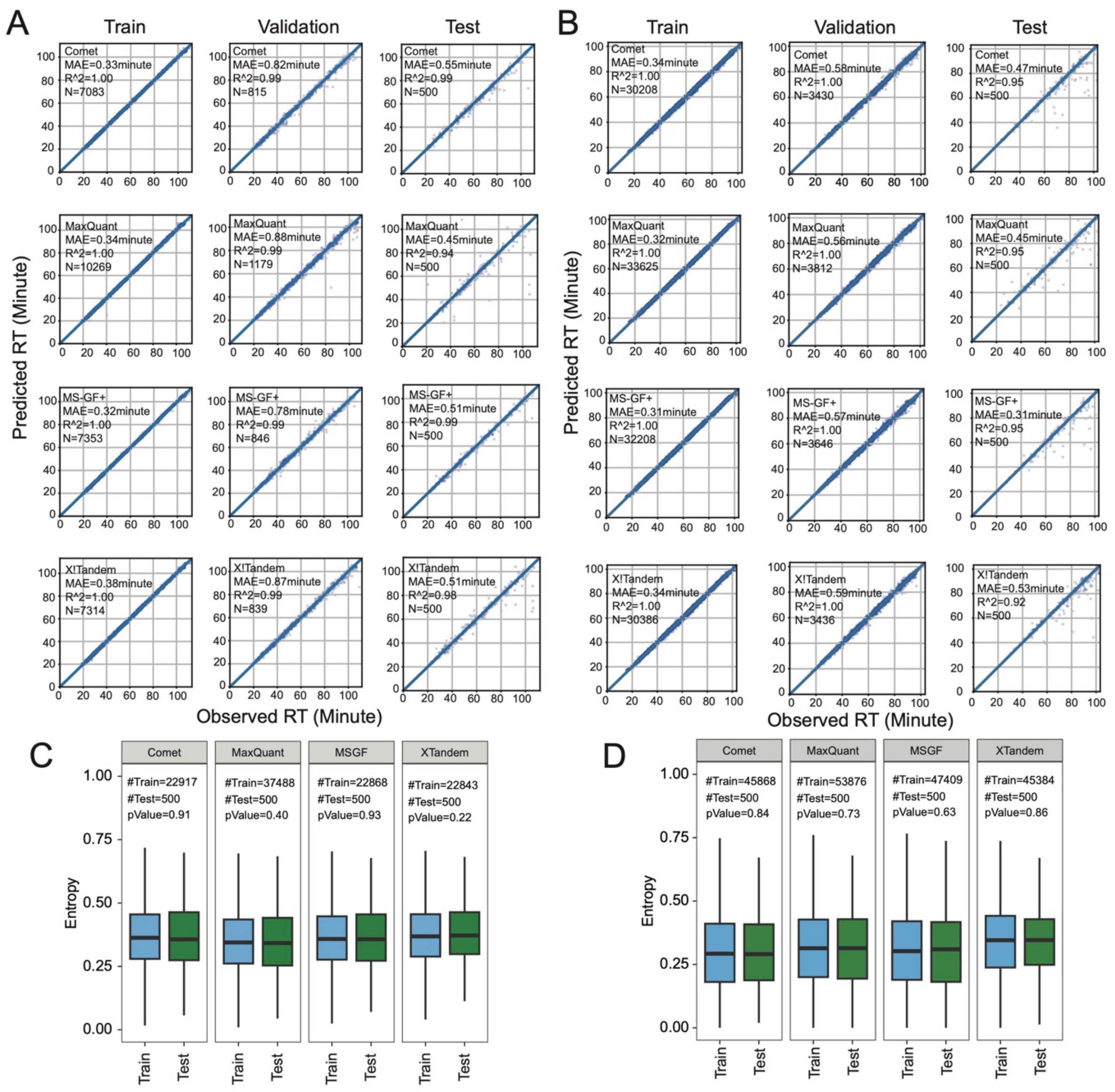

Figure S2: Evaluation of RT prediction model and fragment ion intensity prediction model on both the label free and UCEC TMT datasets. (A) Scatter plots comparing predicted RTs and observed RTs from train, validation and test PSMs of label free datasets of four search engines. (B) Scatter plots comparing predicted RTs and observed RTs from train, validation and test PSMs of UCEC TMT datasets of four search engines. (C) Boxplots comparing predicted fragment ion intensity and observed fragment ion intensity from train and test PSMs of label free datasets of four search engines. p values are based on t-test. (D) Boxplots comparing predicted fragment ion intensity and observed fragment ion intensity from train and test PSMs of UCEC TMT datasets of four search engines. p values are based on t-test.

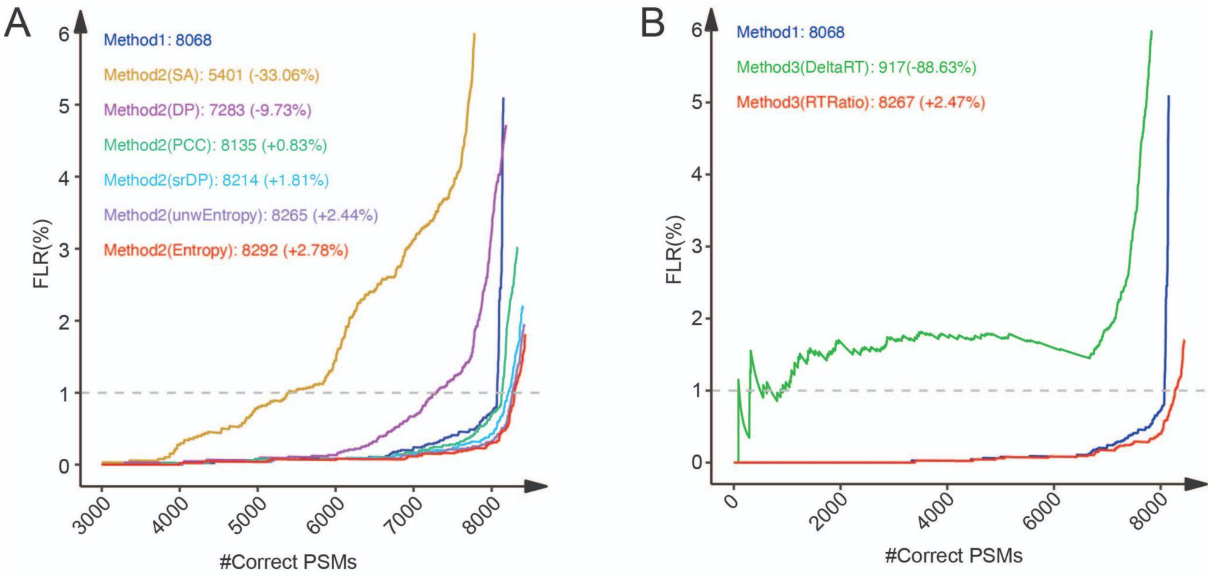

Figure S3: Impact of spectrum similarity (SS) and retention time (RT) difference calculation methods on the performance of site localization on a synthetic phosphopeptide dataset. (A) Impact of six SS calculation methods on the performance of site localization. The number of correctly localized PSMs at different levels of PSM FLR are shown for Method 2 incorporating six different SS calculation methods, respectively. The numbers of correctly localized PSMs at 1% FLR and the percent increase compared with Method 1 (phosphoRS) are indicated. (B) Impact of two RT difference calculation methods on the performance of site localization. The number of correctly localized PSMs at different levels of PSM FLR are shown for Method 3 incorporating two different RT difference calculation methods, respectively. The numbers of correctly localized PSMs at 1% FLR and the percent increase compared with Method 1 (phosphoRS) are indicated.

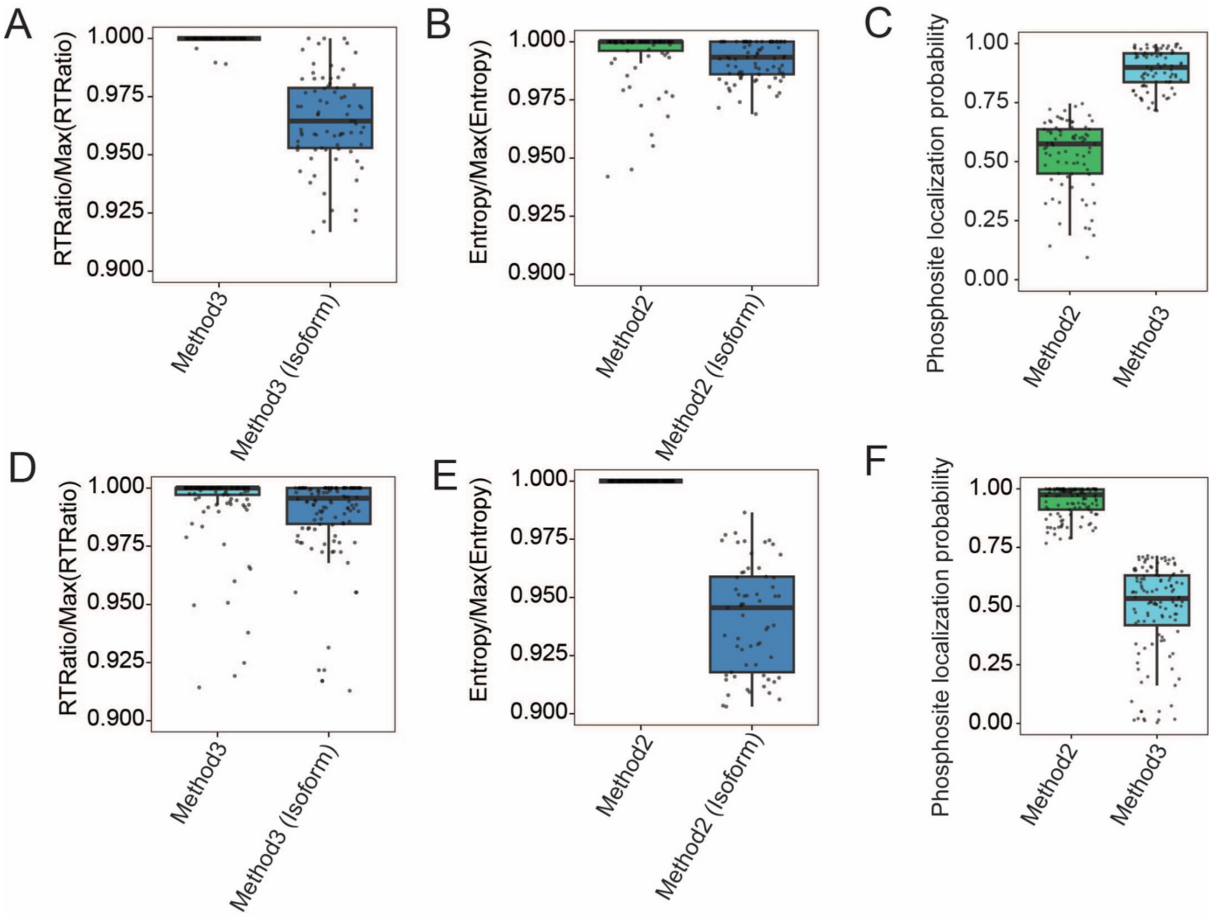

Figure S4: Complementary contributions of RT prediction and fragment ion intensity predictions. (A) Comparison between RT Ratios of the peptide isoforms selected and rejected by Method 3 for the 81 PSMs successfully identified by Methods 3 and 4 but not Methods 1 and 2. (B) Comparison between entropies of the peptide isoforms selected and rejected by Method 2 for the 81 PSMs. (C) Comparison between phosphosite localization probability distributions of Method 2 and Method 3 for the 81 PSMs. (D) Comparison between RT Ratios of the peptide isoforms selected and rejected by Method 3 for the 120 PSMs successfully identified by Methods 2 and 4 but not Methods 1 and 3. (E) Comparison between entropies of the peptide isoforms selected and rejected by Method 2 for the 120 PSMs. (F) Comparison between phosphosite localization probability of Method 2 and Method 3 for the 120 PSMs.

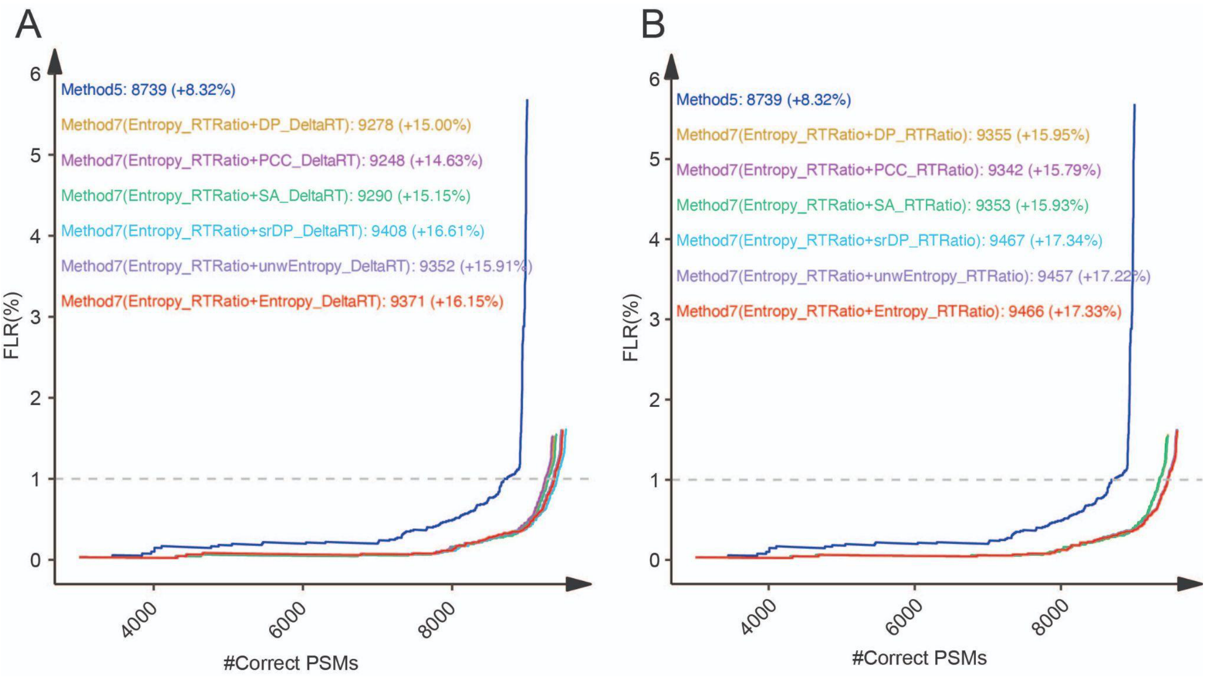

Figure S5: Impact of SS and RT difference calculation methods on the performance of PSM rescoring on a synthetic phosphopeptide dataset. (A) Impact of six SS calculation methods combined with DRT on the performance of PSM rescoring. The number of correctly localized PSMs at different levels of PSM FLR are shown for Method 7 incorporating different SS calculation methods combined with DRT, respectively. The numbers of correctly localized PSMs at 1% FLR and the percent increase compared with Method 5 (phosphoRS+Rescore) are indicated. (B) Impact of six SS calculation methods combined with RTR on the performance of PSM rescoring. The number of correctly localized PSMs at different levels of PSM FLR are shown for Method 7 incorporating different SS calculation methods combined with RTR, respectively. The numbers of correctly localized PSMs at 1% FLR and the percent increase compared with Method 5 (phosphoRS+Rescore) are indicated.

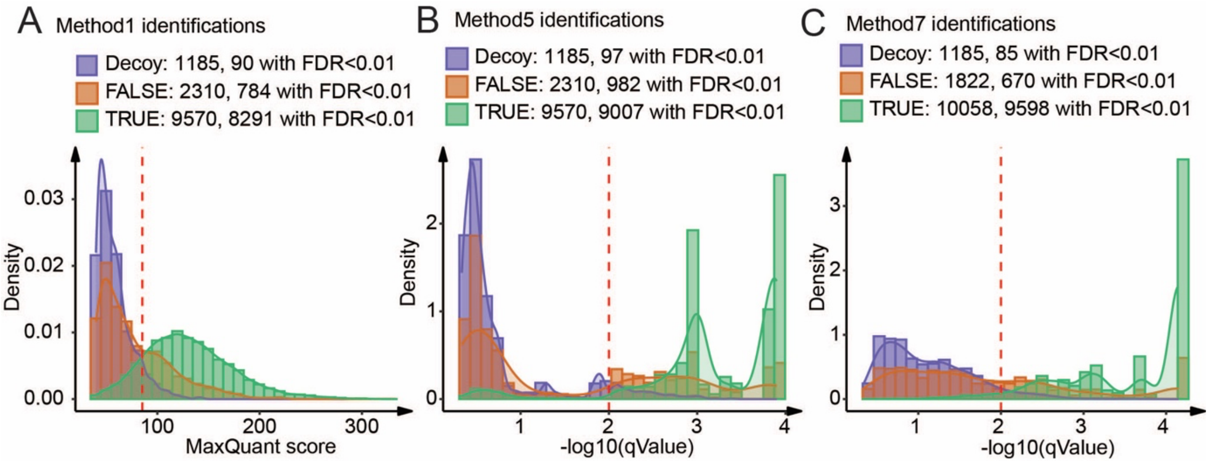

Figure S6: Score histograms of true target, false target, and decoy identifications before and after rescoring on the synthetic dataset. (A) Method 1. (B) Method 5. (C) Method 7. The red dashed lines in each figure represent the 1% FDR cutoff. The value before the comma represents the total number of decoy, false, and true identifications at 100% FDR, respectively. The value after the comma represents the total number of decoy, false, and true identifications at 1% FDR, respectively.

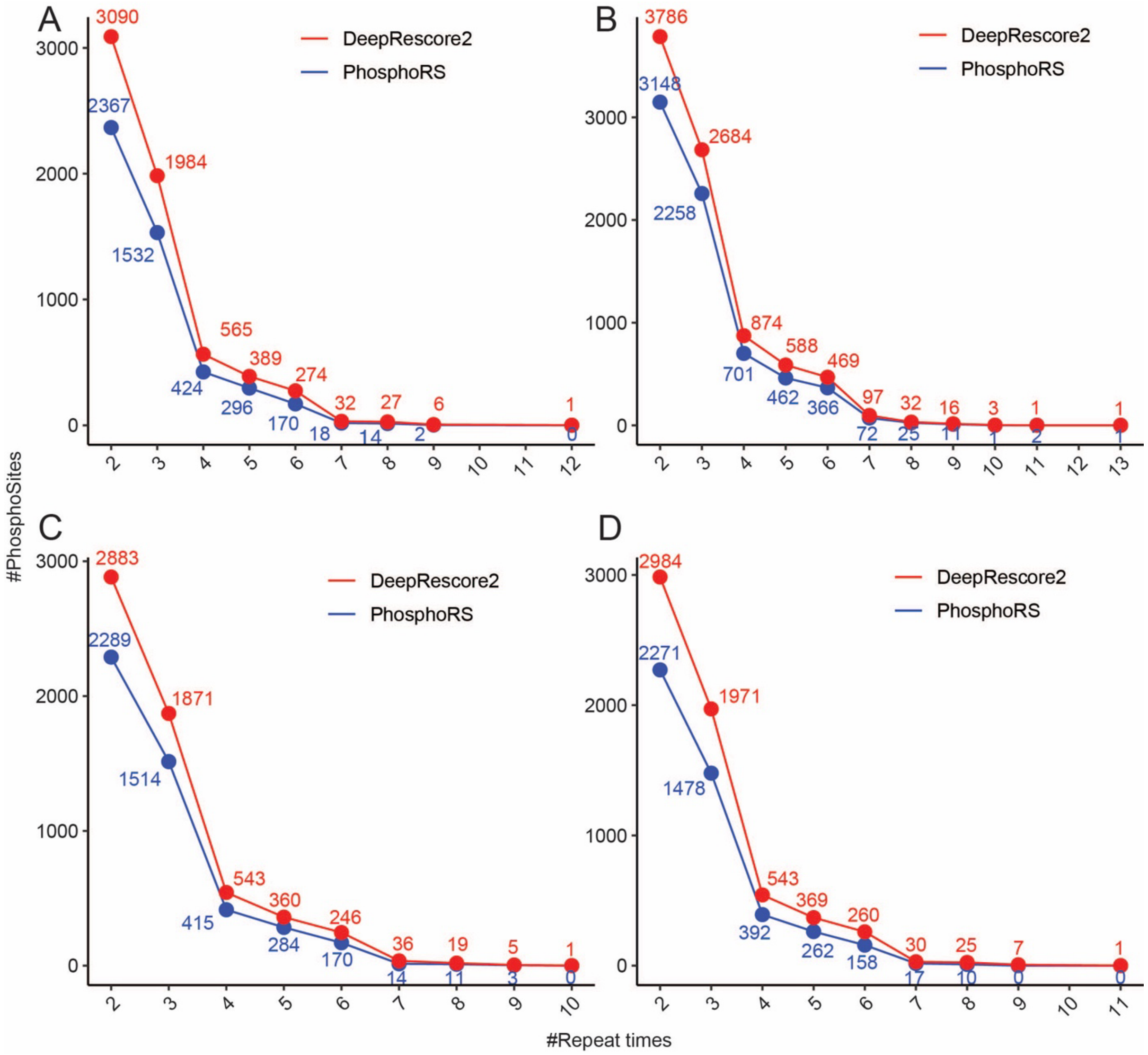

Figure S7: Comparison between PhosphoRS and DeepRescore2 results in the same phospho identification and localization on the label free dataset along an XIC using a 0.5 tolerance window around the RT and mass values of the XIC. (A). Comet. (B) MS-GF+. (C) MaxQuant. (D) X!Tandem.

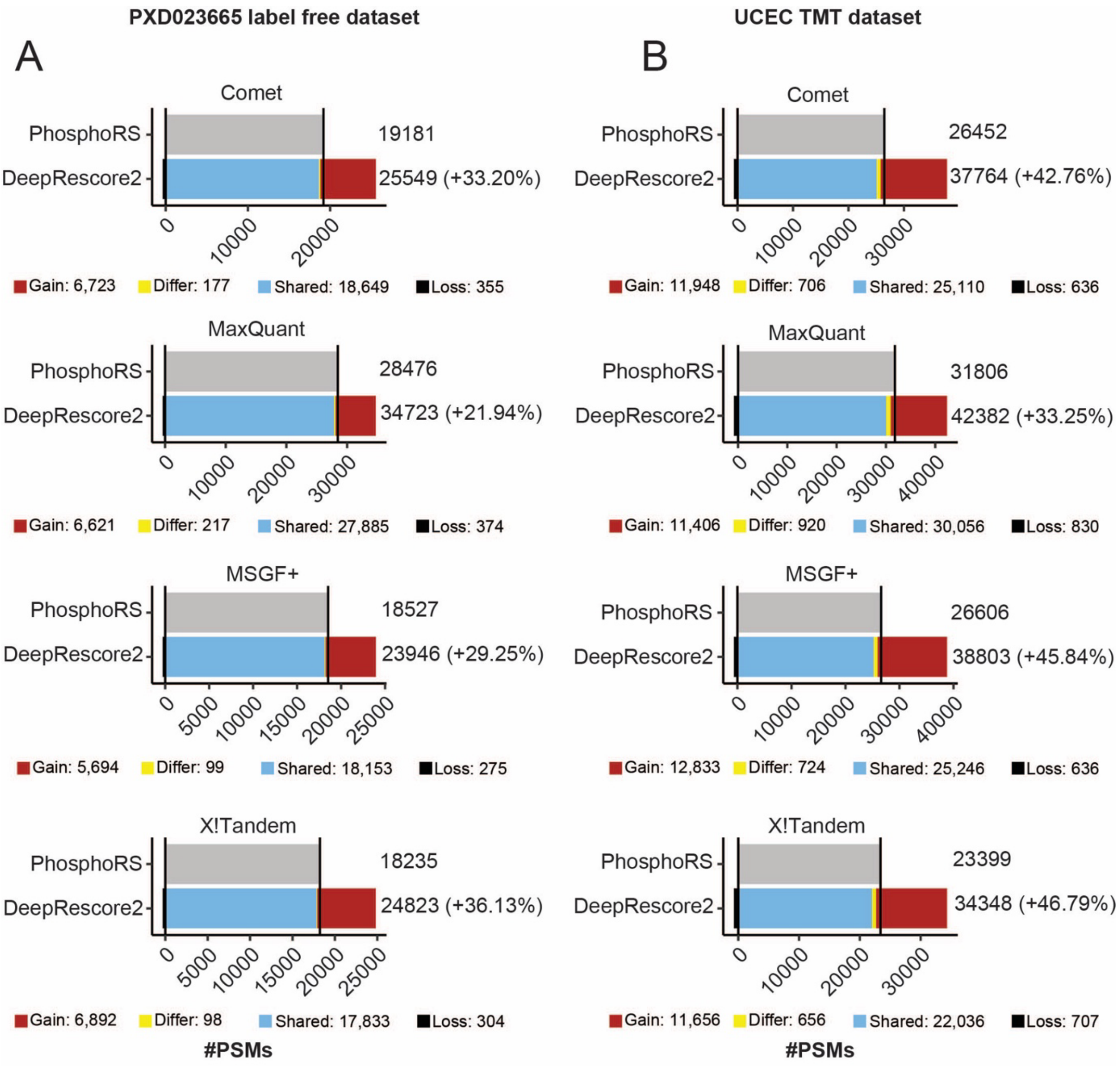

Figure S8: Performance evaluation based on peptide spectrum match (PSM) identifications in two biological datasets using different search engines in combination with PhosphoRS or DeepRescore2. (A) The numbers of PSMs identified from a label-free phosphoproteomic dataset, PXD023665, by four search engines in combination with phosphoRS or DeepRescore2, respectively. (B) The numbers of PSMs identified from the UCEC TMT phosphoproteomic dataset by four search engines in combination with phosphoRS or DeepRescore2, respectively. Gain: PSMs identified by DeepRescore2 but not PhosphoRS. Shared: PSMs identified by both DeepRescore2 and PhosphoRS. Loss: PSMs identified by PhosphoRS but not DeepRescore2.
